# Magnesium Matters: A Comprehensive Review of Its Vital Role in Health and Diseases

**DOI:** 10.7759/cureus.71392

**Published:** 2024-10-13

**Authors:** Ghizal Fatima, Andrej Dzupina, Hekmat B Alhmadi, Aminat Magomedova, Zainab Siddiqui, Ammar Mehdi, Najah Hadi

**Affiliations:** 1 Public Health, Era's Lucknow Medical College and Hospital, Lucknow, IND; 2 Cardiology and Angiology, National Institute of Cardiovascular Diseases, Bratislava, SVK; 3 Biochemistry, College of Medicine, Al-Muthanna University, Samawah, IRQ; 4 Population, Lomonosov Moscow State University, Moscow, RUS; 5 Diabetes and Endocrinology, Era University, Lucknow, IND; 6 Pediatric Dentistry, Career Dental College and Hospital, Lucknow, IND; 7 Medicine, Kufa University, Najaf, IRQ

**Keywords:** deficiency, diseases, health, healthcare, magnesium

## Abstract

Magnesium (Mg), an essential mineral abundantly present within the human body, is intricately involved in a multitude of biochemical processes vital for maintaining health and overall well-being. This review aims to delve into the multifaceted impact of Mg on human health, exploring its physiological functions, dietary sources, and potential health implications of deficiency or insufficiency. Mg plays a pivotal role in various physiological processes, including energy metabolism, muscle contraction, protein synthesis, and DNA synthesis. It acts as a cofactor for more than 300 enzymatic reactions, facilitating the conversion of adenosine triphosphate (ATP) to adenosine diphosphate (ADP) for energy production. Moreover, Mg is essential for the proper functioning of ion channels, particularly calcium channels, influencing nerve transmission and muscle relaxation. Mg is naturally found in a wide array of foods, with green leafy vegetables, whole grains, nuts, seeds, and legumes being particularly rich sources. Additionally, certain fortified foods and dietary supplements provide supplemental Mg intake. Deficiency or insufficiency of mg can have profound implications for health. Inadequate mg levels have been associated with increased risks of various chronic diseases, including hypertension, type 2 diabetes, osteoporosis, and cardiovascular diseases. Furthermore, mg deficiency may manifest as symptoms such as muscle weakness, fatigue, tremors, and irregular heartbeat. Numerous studies have elucidated the relationship between mg intake and the risk of developing chronic diseases. For instance, epidemiological evidence suggests that higher mg intake is associated with a reduced risk of hypertension, possibly due to its vasodilatory effects and influence on blood pressure regulation mechanisms. Similarly, mg has been implicated in the pathophysiology of type 2 diabetes, with mg deficiency contributing to insulin resistance and impaired glucose metabolism. Furthermore, adequate mg intake is crucial for maintaining bone density and reducing the risk of osteoporosis, as mg plays a vital role in bone mineralization and bone health. Understanding the importance of mg in human physiology underscores the significance of ensuring adequate mg intake through diet or supplementation. Healthcare professionals play a critical role in educating individuals about the importance of incorporating mg-rich foods into their diets and considering mg supplementation when necessary, particularly for individuals at risk of deficiency or those with chronic diseases. Mg is an indispensable mineral with far-reaching implications for human health. Its involvement in various physiological processes underscores its importance in maintaining overall health and well-being. Ensuring adequate mg intake is essential for preventing deficiency-related health complications and reducing the risk of chronic diseases. Further research is warranted to elucidate the optimal strategies for mg supplementation and its potential therapeutic applications in disease prevention and management.

## Introduction and background

Magnesium (Mg), an essential mineral vital for human health, is involved in a myriad of physiological processes fundamental to the proper functioning of the body. Its importance extends across multiple organ systems, including the cardiovascular, musculoskeletal, and nervous systems, highlighting its indispensable role in maintaining overall well-being. Despite its significance, Mg deficiency remains a global health concern, with widespread implications for public health. Mg stands as the preeminent divalent intracellular cation within human cells, trailing only potassium (K) in abundance. With an atomic weight of 24.305 g/mol and an atomic number of 12, mg is indispensable for a plethora of biological processes, encompassing oxidative phosphorylation, energy production, glycolysis, and the synthesis of proteins and nucleic acids [1].

Mg is an essential mineral that plays a crucial role in various physiological functions, contributing significantly to overall health and well-being. It is involved in over 300 enzymatic reactions, aiding in energy production, protein synthesis, and DNA and RNA synthesis [2]. One of the most notable health benefits of mg is its impact on cardiovascular health. It helps regulate blood pressure, maintains a normal heart rhythm, and reduces the risk of heart disease by promoting vasodilation and preventing arterial stiffness. Mg also supports bone health by aiding in calcium (Ca) absorption and bone mineralization, which is essential for preventing osteoporosis. Moreover, it has a calming effect on the nervous system, contributing to improved sleep quality and reducing anxiety and depression symptoms. This mineral plays a vital role in muscle function, preventing cramps and spasms by facilitating muscle relaxation. In terms of metabolic health, Mg has been shown to enhance insulin sensitivity, thereby playing a protective role against type 2 diabetes [2].

Additionally, adequate Mg intake is associated with a lower risk of inflammation and may contribute to improved gut health by supporting the growth of beneficial gut bacteria. Overall, mg is vital for maintaining physical and mental well-being. Central to its role is its involvement in mitochondrial adenosine triphosphate (ATP) synthesis, where mg facilitates the formation of MgATP, an essential energy currency within cells. Moreover, Mg's significance extends to cellular signaling, where MgATP serves as a vital substrate for protein phosphorylation and the activation of cyclic adenosine monophosphate (cAMP), a pivotal regulator of diverse biochemical processes [3]. In addition to its roles in energy metabolism and cell signaling, Mg ions play a crucial role in ion transport across cell membranes, muscle contraction, and the modulation of neuronal excitability. The maintenance of cellular Mg homeostasis is intricately linked to the metabolism of other ions, such as K, sodium (Na), and Ca, mediated by mechanisms such as the Na+/K+/ATPase and Ca++-activated K channels [4]. This orchestration of ion fluxes underscores mg's pivotal role in cellular homeostasis and organ function. Mg's physiological significance extends to its involvement in various cellular activities and metabolic pathways, including enzyme-substrate interactions, structural functions, and membrane integrity [5].

Acting as a cofactor in over 600 enzymatic reactions, mg is essential for the activity of protein kinases, glycolytic enzymes, phosphorylation processes, and ATP-dependent reactions [5]. Furthermore, Mg exhibits mild Ca antagonist properties and contributes to numerous structural functions within the cell, including multi-enzyme complexes, G-proteins, protein and nucleic acid synthesis, N-methyl-D-aspartic acid (NMDA) receptors, and mitochondrial function. In recent decades, recognition of Mg's pathophysiological and clinical significance has grown, alongside an understanding of its potential implications in various human diseases [6]. Deficits in Mg have been implicated in the pathogenesis of numerous conditions, prompting further investigation into the therapeutic potential of Mg supplementation and its role in disease prevention and management. This review provides an overview of mg's physiological functions, dietary sources, and the potential health consequences of inadequate intake, setting the stage for a comprehensive exploration of Mg's impact on human health.

## Review

Magnesium metabolism and requirement in the body

Introduction to Magnesium Metabolism

**Figure 1:**
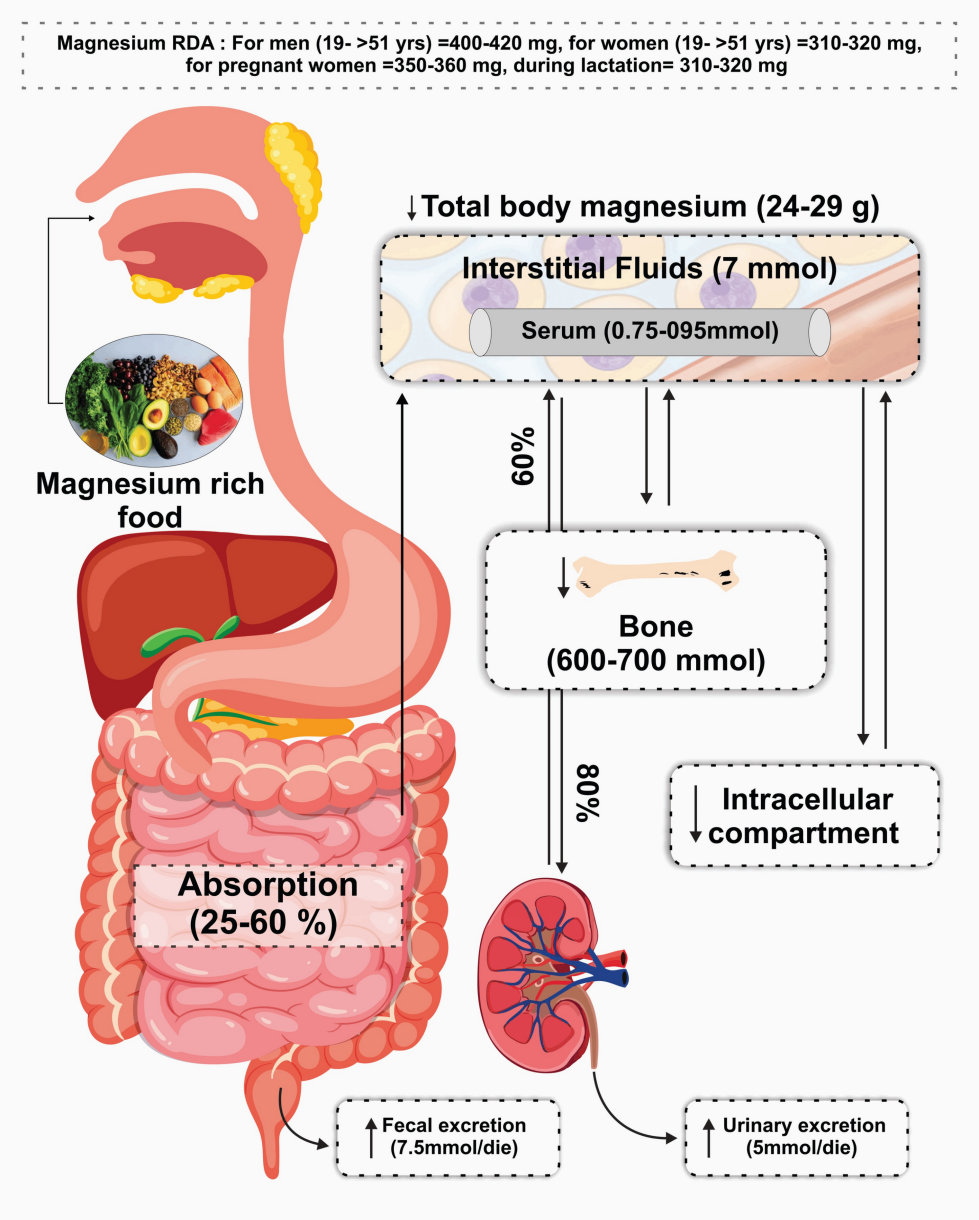
Routes of Mg absorption and excretion Mg, magnesium Credit: authors

Mg metabolism involves absorption, distribution, and excretion, tightly regulated to maintain optimal levels in various tissues and organs (Figure 1). Additionally, understanding the body's Mg requirements is crucial for preventing deficiency and promoting overall health. Mg absorption primarily occurs in the small intestine, predominantly in the ileum and to a lesser extent in the jejunum. Both passive and active transport mechanisms are involved in Mg absorption. Passive paracellular absorption occurs through tight junctions between enterocytes, while active transcellular absorption involves Mg transporters such as TRPM6 (transient receptor potential melastatin 6) and TRPM7, which facilitate Mg uptake into enterocytes. After absorption, Mg is transported via the bloodstream to various tissues and organs.

Distribution of Magnesium in the Body

Mg distribution is regulated by proteins such as albumin and Mg-binding proteins, which help maintain Mg homeostasis. Intracellular Mg levels are tightly regulated by Mg transporters, pumps, and channels located on cell membranes. Notably, the Mg-ATP complex plays a crucial role in intracellular Mg transport and storage, modulating cellular Mg levels [7]. Mg excretion primarily occurs via the kidneys, where it is filtered by the glomeruli and reabsorbed or excreted in the urine. The majority of filtered Mg is reabsorbed in the renal tubules, primarily in the thick ascending limb of the loop of Henle and the distal convoluted tubule. Several transporters, including TRPM6 and TRPM7, regulate Mg reabsorption in the kidneys. Additionally, Mg excretion can occur through feces, sweat, and saliva, although these routes play a minor role compared to renal excretion [8].

Mg homeostasis is tightly regulated by hormonal and non-hormonal mechanisms to maintain optimal intracellular and extracellular Mg levels. Parathyroid hormone (PTH) and calcitriol (active vitamin D) play key roles in Mg homeostasis. PTH increases renal Mg reabsorption in response to low serum Mg levels, while calcitriol enhances intestinal Mg absorption by upregulating the expression of TRPM6 transporters in the small intestine [9]. Mg interacts synergistically with other micronutrients such as Ca, vitamin D, K, zinc, iron, and vitamin B6, highlighting its crucial role in promoting optimal outcomes for both mother and fetus. Insufficient Mg intake during pregnancy has been associated with complications including gestational diabetes, preeclampsia, preterm birth, and low birth weight. Thus, it is important to emphasize maintaining adequate mg levels through a balanced diet and appropriate supplementation [10].

Magnesium Requirements

The Recommended Dietary Allowance (RDA) for Mg varies depending on age, sex, and life stage. The RDA for Mg for adult men is 400-420 mg/day, while for adult women it is 310-320 mg/day. During pregnancy and lactation, Mg requirements increase to support maternal and fetal health. Mg requirements are higher for adolescents, individuals with high physical activity levels, and those with certain medical conditions such as diabetes and hypertension [11]. Mg deficiency can have profound effects on health, leading to symptoms such as muscle cramps, fatigue, weakness, and cardiovascular abnormalities.

Chronic Mg deficiency has been associated with an increased risk of cardiovascular disease (CVD), type 2 diabetes, osteoporosis, and migraine headaches. Therefore, meeting the body's Mg requirements through dietary intake and supplementation when necessary is essential for maintaining optimal health and preventing deficiency-related complications [12]. Mg metabolism involves absorption, distribution, and excretion, tightly regulated by various physiological mechanisms. Understanding the body's Mg requirements is crucial for maintaining Mg balance and preventing deficiency-related health issues. Adequate dietary intake of Mg-rich foods and supplementation when necessary can help meet the body's Mg needs and promote overall health and well-being.

Magnesium deficiencies associated with reduced magnesium intake

Causes of Reduced Magnesium Intake

Mg deficiencies often stem from inadequate dietary intake, a prevalent issue worldwide. Studies consistently demonstrate that dietary Mg intake falls short of recommended levels, with a significant proportion of individuals consuming Mg below the recommended daily allowance (RDA). Western diets, characterized by high consumption of refined foods and low intake of Mg-rich whole grains and green vegetables, contribute to this shortfall. Processing and cooking methods further deplete the Mg content in food, exacerbating the problem. Additionally, factors such as pathogenic gut microbiota, phytic acid in certain foods, and the use of pesticides like glyphosate can interfere with Mg absorption, compounding the issue of deficiency. The consequences of Mg deficiency are multifaceted and can manifest as muscle cramps, fatigue, weakness, cardiovascular abnormalities, and increased risk of chronic diseases such as CVD, type 2 diabetes, and osteoporosis.

Clinical Manifestations of Magnesium Deficiency

Addressing Mg deficiency requires attention to dietary habits, supplementation when necessary, and consideration of factors affecting Mg absorption. Overall, recognizing the association between reduced Mg intake and deficiency-related health risks underscores the importance of promoting adequate Mg consumption for optimal health and well-being. Numerous studies have consistently highlighted the inadequate dietary Mg intake prevalent in Western countries, often falling below recommended daily levels [13,14]. A recent study reported that nearly two-thirds of Americans consume Mg below the RDA, with a substantial portion ingesting less than 75% of the RDA, and a concerning 19% falling below 50% of the RDA [15].

This pattern is mirrored in Europe, where even physically active and well-educated individuals fail to meet dietary Mg recommendations [16]. Processing and cooking methods further deplete Mg content, with boiling particularly notorious for Mg loss [17]. Diets, characterized by an abundance of refined foods and a deficiency of Mg-rich whole grains and green vegetables, contribute to this shortfall [18]. Moreover, pathogenic gut microbiota may interfere with Mg absorption by converting trans-aconitate to tricarballylate, a compound that chelates Mg, reducing its availability [19,20]. Additionally, phytic acid found in certain foods can hinder Mg absorption, while glyphosate, a commonly used pesticide, further diminishes soil Mg levels [21].

However, organic farming practices have demonstrated higher Mg content in produce compared to conventional methods [22]. Mg serves as a common additive in various food products and pharmaceutical formulations, playing roles such as an anticaking agent and a preservative [23]. Drinking water, particularly mineral-rich varieties, represents a supplementary source of Mg intake. Individuals consuming Mg-rich water exhibit higher Mg intake levels than those drinking low-mineralized or tap water, although the Ca-to-Mg ratio in water may influence absorption [24].

Long-Term Health Consequences

The bioavailability of Mg in drinking water surpasses that in food, presenting an advantageous avenue for Mg supplementation. While adding Mg to water is feasible, fortifying foods with Mg is challenging. Furthermore, the Mg content of water used in cooking can impact the nutrient content of prepared meals, potentially reducing Mg loss during the cooking process. With the increasing global scarcity of fresh water, the widespread adoption of desalinated seawater (DSW) for drinking poses concerns regarding Mg intake [25]. Desalination removes Mg from water, potentially exacerbating hypomagnesemia, which has been linked to heightened cardiac morbidity and mortality [26]. In regions heavily reliant on DSW for drinking water, addressing Mg deficiency becomes a critical public health consideration.

Deficit of magnesium associated with aging

Physiological Changes in Aging

As individuals age, they often experience changes in Mg metabolism that can contribute to deficiencies in this vital mineral. Several factors contribute to the deficit of Mg associated with aging. First, aging is often accompanied by alterations in gastrointestinal function, including reduced absorption of Mg from the diet. Age-related changes in stomach acid production and intestinal motility can impair the absorption of Mg, leading to lower levels of this mineral available for bodily functions. Furthermore, older adults may have diets that are inadequate in Mg-rich foods. Poor dietary choices, decreased appetite, or restricted diets due to medical conditions can all contribute to lower Mg intake in older individuals [27]. Additionally, aging is associated with increased loss of Mg through urine and sweat, further exacerbating Mg deficiency. Changes in kidney function and hormonal regulation can impact Mg excretion, leading to greater losses of this mineral from the body.

Impact of Magnesium Deficiency on Elderly Health

The consequences of Mg deficiency in aging individuals can be significant. Low Mg levels have been linked to a variety of age-related health issues, including CVD, osteoporosis, muscle weakness, and cognitive decline. Aging often brings about a total body Mg deficit [18]. Despite this, serum Mg levels typically remain constant with age, with alterations in serum Mg often linked to underlying diseases or changes in kidney function [28]. In healthy older individuals, studies have shown an age-dependent decrease in cellular Mg concentration, even in the absence of significant changes in total serum Mg levels [29].

Chronic latent Mg deficiency is a common occurrence among older adults in Western countries [29]. Several factors contribute to this demonstrated Mg insufficiency with aging. Notably, this Mg shortfall is frequently associated with low Mg intake, despite the fact that Mg requirements for bodily processes do not typically change with age [30-32]. The intestinal absorption of Mg tends to decline with age, which may contribute to Mg deficits in aging individuals [33]. This decline in absorption is often exacerbated by disturbances in vitamin D homeostasis, which are common in older adults. Furthermore, renal reabsorption of Mg, primarily occurring in the loop of Henle and proximal convoluted tubule, may be compromised due to reduced kidney function, a prevalent issue in the elderly.

Strategies for Mitigating Magnesium Deficits in the Aging Population

Secondary mg deficiencies in older adults can be attributed to various factors, including the presence of certain medical conditions and the use of multiple medications [34]. Diuretic therapy, commonly prescribed in older adults, can lead to excessive urinary loss of mg. Diuretic-induced hypomagnesemia often accompanies hypokalemia, with mg correction being necessary for K balance. Therefore, assessing mg levels in patients with hypokalemia is advisable. Moreover, several medications commonly used in the elderly, such as antacids, H2 blockers, proton pump inhibitors, antihistamines, antibiotics, antiepileptic drugs, and antivirals, among others, may contribute to mg deficits. Addressing mg deficiency in aging populations requires a multifaceted approach. A diet rich in mg-containing foods, considering mg supplementation when necessary, and addressing factors that may impair mg absorption or increase excretion are all important strategies for maintaining optimal mg status and promoting healthy aging. To mitigate Mg deficits in the aging population, several strategies can be implemented. First, encourage dietary adjustments that include Mg-rich foods such as leafy greens, nuts, seeds, whole grains, and legumes. Fortifying foods with Mg can also help meet daily requirements. Additionally, consider recommending Mg supplements, particularly for those with difficulty absorbing nutrients or specific medical conditions. Regular health check-ups can identify deficiencies early, enabling timely interventions. Furthermore, educating seniors on the importance of maintaining adequate Mg levels for bone health, cardiovascular function, and overall well-being can foster better dietary choices and adherence to supplementation.

Magnesium, inflammation, and oxidative stress

Role of Magnesium in Modulating Inflammatory Responses

Low levels of Mg have been consistently associated with inflammation and oxidative stress. Mg deficiency can lead to increased production of reactive oxygen species (ROS) and impaired antioxidant defense mechanisms, contributing to oxidative stress. Additionally, Mg deficiency has been linked to elevated levels of proinflammatory cytokines and markers of inflammation. Studies have demonstrated that inadequate Mg intake or low serum Mg levels are correlated with higher levels of inflammation markers such as C-reactive protein (CRP), interleukin-6 (IL-6), tumor necrosis factor-alpha (TNF-alpha), and vascular cell adhesion molecule-1 (VCAM-1).

Moreover, Mg plays a crucial role in regulating cellular signaling pathways involved in inflammation and oxidative stress. Therefore, maintaining adequate Mg levels through dietary intake or supplementation may help mitigate inflammation and oxidative stress, potentially reducing the risk of chronic diseases associated with these conditions. Mg deprivation, low serum Mg levels, and reduced dietary Mg intake have all been consistently associated with increased production of oxygen free radicals, low-grade systemic inflammation, and elevated levels of inflammation markers and proinflammatory molecules in preclinical, epidemiological, and clinical human studies [35-42]. Kramer and co-workers observed the highest substance P production in rats fed with high Mg-rich feed (RDA:100-105%).

Magnesium’s Impact on Oxidative Stress and Cellular Health

**Figure 2:**
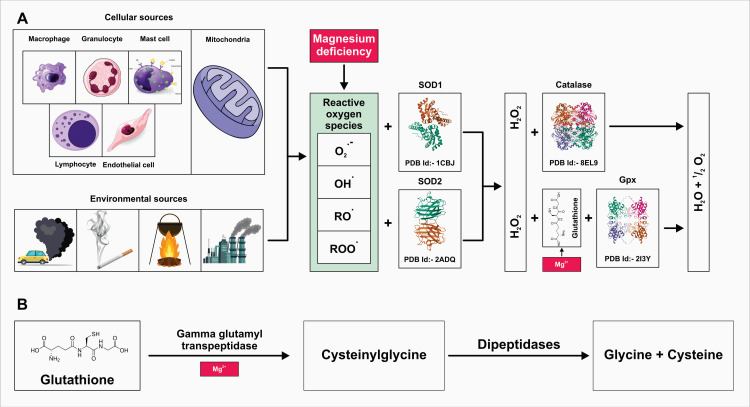
Illustrating the cellular and environmental sources of Mg deficiency and its consequences, particularly the generation of ROS Cellular and environmental sources of Mg deficiency and its consequences, in the generation of ROS, and the role of antioxidants like glutathione in mitigating oxidative stress. A: cellular and environmental sources of MG deficiency and ROS generation, B: highlights the role of antioxidants like glutathione, as well as the precursor molecules cysteinylglycine, glycine, and cysteine, in combating oxidative damage and maintaining cellular health in the face of Mg deficiency. Mg, magnesium; ROS, reactive oxygen species Credits: authors

Mg plays a crucial role in mitigating oxidative stress by acting as a cofactor for antioxidant enzymes. Adequate Mg levels support the body's defense mechanisms against free radicals, helping to maintain cellular integrity and function. Deficiency in Mg can lead to increased oxidative stress, which is associated with various chronic diseases, including CVD, diabetes, and neurodegenerative disorders. By regulating mitochondrial function and energy metabolism, Mg also helps prevent cellular damage caused by oxidative stress. Therefore, maintaining optimal Mg levels is essential for promoting cellular health and reducing the risk of oxidative stress-related conditions. Substance P is a known regulator of anti-oxidant enzymes and is produced in response to early inflammatory events in cells [39]. Furthermore, the authors found a reduced concentration of plasma glutathione in low Mg-fed rats [39]. Chou et al. observed an inverse relationship between dietary Mg intake and CRP levels [15]. Similarly, Xu et al. found that serum Mg levels were negatively associated with obesity and abdominal obesity in type 2 diabetes mellitus (T2DM) [42]. Mg depletion leads to increased production of oxygen-derived free radicals (ROS), oxygen peroxide, and superoxide anion by inflammatory cells (Figure 2).

Additionally, Mg deficiency not only heightens oxidative stress but also diminishes antioxidant defense competence [35,43]. Mg is essential for the proper functioning of gamma-glutamyl transpeptidase, a key enzyme involved in glutathione synthesis, suggesting a potential mild antioxidant action of Mg [44,45]. In humans, a correlation between intracellular Mg and the circulating reduced/oxidized glutathione ratio has been observed [46]. Another study reported a negative correlation between Mg levels and oxidative stress markers (plasma superoxide anions and malondialdehyde) in a population exposed to chronic stress [47]. Aging is often accompanied by a low-grade inflammatory state termed "inflammaging" [48]. It has been proposed that chronic Mg insufficiency may facilitate this inflammaging condition and impair redox status, thereby contributing to the development of age-related illnesses [19,49]. Specifically, a link has been suggested between Mg inadequacy and the occurrence of insulin resistance, T2DM, and cardiometabolic syndrome.

Role of magnesium in immune response

Magnesium’s Influence on Immune Cell Function

Mg influences immune cell function by enhancing T cell activation, promoting macrophage activity, and regulating inflammatory responses critical for immune defense. Mg, often overlooked in discussions about immune health, plays a critical role in regulating the body's immune response. Emerging research suggests that Mg deficiency can compromise various aspects of immune function, leaving individuals more susceptible to infections and inflammatory conditions. Mg influences both the innate and adaptive arms of the immune system, modulating the activity of immune cells such as macrophages, T cells, and B cells [50]. One of Mg's key functions is its involvement in the production and function of antibodies, which are essential for recognizing and neutralizing pathogens. Additionally, Mg acts as a cofactor for enzymes involved in DNA replication and repair, crucial processes for mounting an effective immune response. Moreover, Mg helps regulate the production of cytokines, the signaling molecules that coordinate immune cell communication and inflammation. Mg plays a pivotal role in modulating acquired immunity by influencing the proliferation and development of lymphocytes [51].

Magnesium homeostasis

The transient receptor potential cation channel, subfamily M, member 7 (TRPM7), crucial for Mg homeostasis in immune cells, regulates cytosolic Mg levels. Studies on TRPM7-deficient B cell lines revealed a decrease in free cytosolic Mg and cell cycle arrest, which was mitigated by a high Mg culture medium; additionally, impaired T cell development was observed in TRPM7 knockout mice [52]. Furthermore, Mg deficiency may expedite thymus involution, as evidenced by increased apoptosis in thymus from Mg-deficient rats [53]. Mg deficiency alters polymorphonuclear cell number and functionality and activates phagocytosis [38]. Additionally, Mg is involved in regulating cell apoptosis, as demonstrated in Fas-induced β-cell apoptosis, where elevated cellular free Mg levels are necessary for initiating cellular apoptosis and death [54].

Mitochondria are the source of elevated intracellular Mg, which later on initiates apoptotic events followed by DNA fragmentation and phosphatidylserine externalization [55]. Moreover, Mg plays a crucial role in the synthesis, transport, and activation of vitamin D, an important immunomodulator in infectious diseases such as SARS-CoV-2 infection [37]. Mg deficit may contribute to immune hyperresponsiveness, cytokine storm, endothelial dysfunction, thrombotic complications, and worsened prognosis in COVID-19, particularly in individuals with predisposing conditions like old age, diabetes, and hypertension. Research suggests that maintaining optimal Mg levels may enhance immune function and support overall health. However, many individuals, particularly older adults and those with certain medical conditions, may be at risk of Mg deficiency. Therefore, understanding the importance of Mg in immune health and ensuring adequate intake through diet or supplementation is essential for bolstering immune resilience and promoting wellness.

Magnesium and cardiovascular diseases

Magnesium Deficiency and Cardiovascular Risk Factors

Mg plays a crucial role in maintaining cardiovascular health, and its deficiency has been implicated in the pathogenesis of hypertension and CVDs. Epidemiological studies have consistently demonstrated an inverse association between dietary Mg intake and the risk of developing hypertension and CVDs.

Mechanisms of Magnesium in Cardiovascular Health

One of the mechanisms through which mg influences blood pressure regulation is its vasodilatory effect. Mg supports cardiovascular health by regulating vascular tone, enhancing endothelial function, reducing arterial stiffness, and modulating Ca levels, which collectively contribute to lower blood pressure and improved heart function. Mg acts as a natural Ca channel blocker, promoting smooth muscle relaxation and vasodilation, which helps to lower blood pressure. Additionally, Mg regulates endothelial function and nitric oxide (NO) production, essential factors in vascular health and blood pressure regulation. Furthermore, mg deficiency has been linked to endothelial dysfunction, inflammation, oxidative stress, and insulin resistance, all of which contribute to the development and progression of hypertension and CVDs [55]. CVD is believed to be an outcome of such events. The regions with hard water, rich in Mg and Ca, had lower cardiovascular death rates and stroke incidence [56]. Theisen confirmed this, showing significantly lower cardiovascular death rates in areas with hard water compared to those with soft water [57].

Mg plays a crucial role in blood pressure regulation by modulating vascular tone and contractility through its influence on Ca levels [58-60]. It acts as a natural Ca channel blocker, modulating Ca-channel activity in heart cells [61,62]. Mg deficiency stimulates aldosterone synthesis, thromboxane, and soluble intercellular adhesion molecule 1trictor prostaglandin production [63]. Additionally, Mg has a beneficial effect on vascular endothelium, influencing the release of NO and prostacyclin [64]. Oral Mg supplementation has been shown to improve endothelial function in older adults with T2DM [65].

In hypertensive disorders, Mg deficits may contribute to vascular hyper-reactivity and elevated blood pressure [66]. While serum total Mg levels are typically normal in hypertensive individuals, defects in Mg homeostasis have been documented [67]. Epidemiological studies have shown an inverse relationship between dietary Mg intake and blood pressure [29,68]. Intracellular free Mg concentrations are lower in hypertensive individuals compared to normotensive controls, and altered Mg urinary excretion has been observed in hypertensive individuals [69,70].

Clinical Implications and Magnesium Supplementation

Early studies suggested Mg therapy for lowering blood pressure in patients with malignant hypertension [71,72]. Intravenous Mg has shown consistent benefits in preeclampsia, eclampsia, and malignant hypertension [73,74]. However, the response to oral Mg supplements in essential hypertension is less clear [75-78]. While some studies suggest hypotensive effects of Mg supplementation, others show no effect or even a potential worsening of blood pressure [76,78]. Nevertheless, meta-analyses and systematic reviews support the key role of Mg in hypertension, emphasizing the inverse relationship between dietary Mg intake and hypertension prevalence and incidence [55].

**Figure 3:**
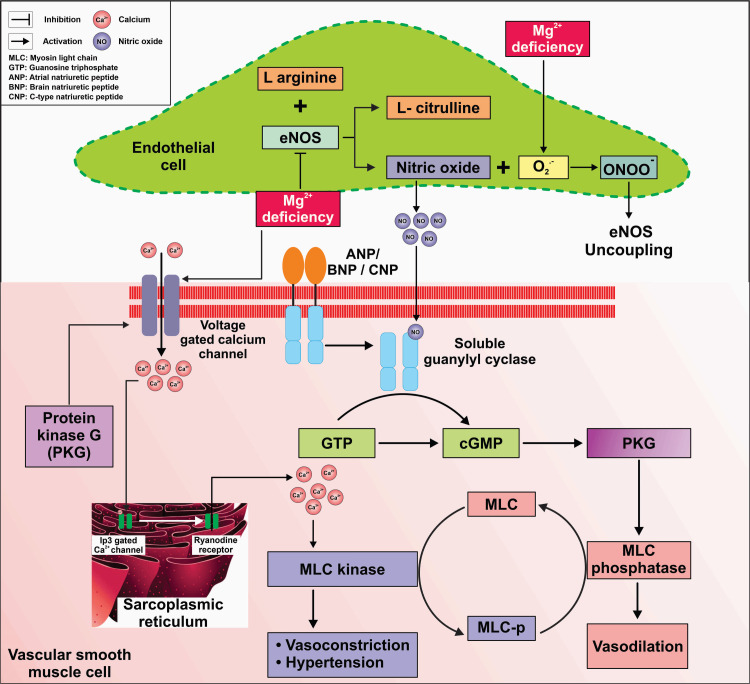
Mg deficiency disrupts the function of eNOS, reducing NO production from its substrate L-arginine. Decreased NO availability impairs vasodilation Mg deficiency may inhibit PKG, which normally promotes the relaxation of smooth muscle cells by inhibiting Ca channels and MLC kinase. This leads to increased Ca influx and MLC phosphorylation, causing vasoconstriction and elevated blood pressure. Mg, magnesium; Ca, calcium; eNOS, endothelial nitric oxide synthase; PKG, protein kinase G; MLC, myosin light chain Credit: authors

Endothelial dysfunction is marked by the decreased production of NO, a potent vasodilator in endothelial cells (Figure 3). Mg is a known accelerator of endothelial NO synthase enzyme and its deficiency increases the reactive nitrogen species production in response to low NO bioavailability [55,64]. Mg serves as an antagonist of voltage-gated Ca channels, which are responsible for the induction of Ca-induced vasoconstriction of smooth muscle cells [71]. Therefore, Mg deficiency disrupts the intricate balance of vasodilation and vasoconstriction in the vascular system, ultimately leading to hypertension [55,66,71].

Mg supplementation has shown promising results in improving endothelial function, reducing inflammation, and lowering blood pressure in both hypertensive and normotensive individuals. Moreover, Mg plays a vital role in cardiac muscle function and rhythm regulation. Adequate Mg levels are necessary for maintaining normal myocardial contractility and electrical stability [71]. Mg deficiency can lead to arrhythmias, including atrial fibrillation and ventricular tachycardia, and increase the risk of myocardial infarction and sudden cardiac death.

Several studies have highlighted the beneficial effects of Mg supplementation in reducing the risk of CVD events and mortality. However, further research is needed to elucidate the optimal dosage and duration of Mg supplementation for cardiovascular protection. Mg deficiency is associated with an increased risk of hypertension and CVDs through various mechanisms, including vasomotor dysfunction, endothelial dysfunction, inflammation, oxidative stress, and arrhythmias. Therefore, ensuring adequate Mg intake through diet or supplementation may represent a valuable strategy for the prevention and management of hypertension and CVDs.

Magnesium and Type 2 Diabetes

Mg plays a crucial role in the pathophysiology of T2DM. Epidemiological studies have consistently shown an inverse relationship between Mg intake and the risk of developing T2DM. Mg deficiency has been associated with insulin resistance, impaired glucose metabolism, and decreased insulin sensitivity, all hallmark features of T2DM. Mechanistically, Mg is involved in glucose transport and utilization, insulin signaling, and pancreatic beta-cell function. Mg acts as a cofactor for enzymes involved in glucose metabolism and insulin action, facilitating glucose uptake into cells and promoting insulin-mediated glucose utilization.

Association Between Magnesium Levels and Insulin Sensitivity

Higher mg levels are linked to improved insulin sensitivity, which helps regulate blood sugar levels and reduces the risk of type 2 diabetes. Additionally, mg modulates insulin receptor activity and intracellular signaling pathways involved in glucose metabolism. Furthermore, mg deficiency exacerbates chronic low-grade inflammation and oxidative stress, contributing to the progression of insulin resistance and T2DM complications. Clinical studies have demonstrated that mg supplementation improves insulin sensitivity, glycemic control, and metabolic parameters in individuals with T2DM or at risk of developing the disease. A substantial body of evidence has established a link between mg deficiency and alterations in insulin sensitivity, as well as the development of T2DM [79-81]. Despite normal total serum Mg levels, patients with T2DM often exhibit lower cellular and/or ionized plasma Mg concentrations [82,83]. Potential mechanisms contributing to Mg depletion in T2DM include low dietary Mg intake and increased Mg urinary loss, with absorption and retention of dietary Mg remaining unchanged [84]. Hyperglycemia and hyperinsulinemia in T2DM have been implicated in excessive urinary Mg excretion, while insulin resistance may disrupt Mg transport [85,86].

These disruptions in Mg metabolism may predispose individuals to T2DM development and impair insulin-mediated glucose uptake. Given this evidence, Mg supplementation has been proposed as a non-pharmacologic, cost-effective, and safe approach for preventing and managing T2DM. However, prospective trials investigating the effects of Mg supplementation in individuals with or at risk of T2DM are limited [87,88]. While several studies have demonstrated modest improvements in glycemic profiles with Mg supplementation, not all trials have shown consistent results. A systematic review and meta-analysis of 18 double-blind randomized controlled trials found that Mg supplementation may have beneficial effects on glucose parameters in individuals with T2DM and improve insulin-sensitivity parameters in those at high risk of T2DM [89]. Furthermore, recent research utilizing an umbrella review to assess health outcomes associated with Mg intake and supplementation has confirmed the inverse association between elevated Mg intake and the risk of T2DM [78]. Maintaining adequate Mg levels through dietary intake or supplementation may play a critical role in preventing and managing T2DM by improving insulin sensitivity, glucose metabolism, and overall metabolic health.

Magnesium and Respiratory Diseases

Mg has garnered significant attention in the realm of respiratory diseases due to its potential therapeutic implications. Research suggests a multifaceted role for Mg in various respiratory conditions, ranging from asthma to chronic obstructive pulmonary disease (COPD) and acute respiratory distress syndrome (ARDS). In asthma, Mg has demonstrated broncho-dilatory effects, with intravenous Mg sulfate administration showing promise in acute exacerbations by enhancing bronchial smooth muscle relaxation.

Additionally, Mg supplementation may mitigate airway hyperresponsiveness and improve lung function parameters in asthmatic individuals. In 1940, Rovsing proposed the potential of Mg in managing asthma, observing favorable clinical outcomes following intravenous Mg sulfate administration in two hospitalized patients experiencing acute asthma exacerbations [90]. Subsequent reports throughout the decades corroborated these findings, demonstrating positive responses to Mg treatment in acute airway constriction episodes, suggesting a potential broncho-dilatory effect of Mg [91,92]. However, conflicting results emerged from other studies, failing to confirm the therapeutic efficacy of Mg in asthma management [93,94]. Interestingly, intravenous Mg administration appears to enhance the broncho-dilatory effects of medications like terbutaline and salbutamol, further improving pulmonary function tests [95,96].

Mg regulates the contractile state of bronchial smooth muscle cells, with Mg depletion triggering bronchial constriction while Mg restoration induces relaxation. Multiple mechanisms have been proposed to explain the positive effects of Mg on bronchial smooth muscle relaxation, including its Ca channel-blocking properties, reduced sensitivity to acetylcholine-induced depolarization, stabilization of mast cells and T-lymphocytes, and stimulation of NO and prostacyclin production [97-99].

Clinical Implications and Magnesium Supplementation

Mg supplementation may offer clinical benefits for individuals with type 2 diabetes by improving insulin sensitivity, enhancing glycemic control, and reducing complications. Studies suggest that adequate mg intake can help lower blood sugar levels and support overall metabolic health, making it a valuable addition to diabetes management strategies. Regular monitoring is recommended. In the general population, higher dietary Mg intake has been associated with improved lung function, decreased airway reactivity, and respiratory symptoms such as wheezing, suggesting a potential role of low Mg intake in asthma pathogenesis [100]. However, serum total Mg levels have limited clinical utility in asthma management, as no significant differences have been observed between asthmatic and non-asthmatic individuals during acute exacerbations. Instead, alterations in cellular Mg levels, reflecting overall body Mg status, have been implicated in asthma, with lower levels correlating with increased bronchial reactivity [101,102]. Consequently, non-pharmacological Mg supplementation may offer a complementary approach to managing asthma by addressing cellular Mg deficits and modulating bronchial smooth muscle reactivity [103,104].

Magnesium and psychiatric disorders

Magnesium Deficiency and Its Impact on Mental Health

Mg has garnered interest in psychiatric disorders due to its potential role in modulating neurotransmission, mood regulation, and stress response. Research suggests that Mg deficiency may be associated with conditions such as depression, anxiety, and schizophrenia. Supplementation with Mg has shown promise in alleviating symptoms and improving mental well-being in some individuals. However, the precise mechanisms underlying Mg's effects on psychiatric disorders remain to be fully elucidated, and further research is needed to clarify its therapeutic potential and optimize its use as an adjunctive treatment in psychiatric care.

Mechanisms Linking Magnesium to Neurotransmitter Regulation

Mg plays a vital role in neurotransmitter regulation by influencing the synthesis and release of serotonin and dopamine, enhancing neuronal function, and protecting against excitotoxicity, which affects mood and cognition. Several psychiatric disorders, including anxiety, depression, irritability, insomnia, hypochondriasis, panic attacks, hyperexcitability, headache, dizziness, tremors, and psychotic behavior, have been associated with Mg deficiency [105]. Neuromuscular symptoms such as asthenia, muscular weakness, and myalgias, as seen in chronic fatigue syndrome and fibromyalgia, may also arise. Enzymes and cellular reactions involved in stress responses are Mg-dependent.

Reduced serum Mg levels have been proposed in subjects with depression, with a recent study by Noah et al. indicating latent Mg insufficiency in nearly half of patients screened for stress [106,107]. Mg deficit may induce electrophysiological evidence of hyperexcitability in the central nervous system (CNS). Alterations in electroencephalogram (EEG) activity during auditory stimuli have been observed in Mg-deficient rats, suggesting CNS hyperexcitability [108,109]. In humans, Mg insufficiency has been linked with neuro-muscular hyperexcitability.

Various mechanisms may link Mg deficiency to nervous hyperexcitability, including Mg's modulatory actions on cellular Ca and neurotransmitters [105]. Mg supplements have been proposed as helpful in depression treatment due to their modulatory role on NMDA-receptor complex ion channels [110,111]. Higher Mg intakes have been associated with reduced depression symptoms [112]. Some antidepressant drugs like sertraline and amitriptyline may increase intracellular Mg levels [113].

Therapeutic Potential of Magnesium Supplementation in Psychiatric Treatment

Mg supplementation shows promise as a complementary treatment for psychiatric disorders, potentially alleviating symptoms of anxiety and depression. Research indicates that adequate Mg levels can enhance treatment efficacy, improve mood, and support cognitive function. Integrating Mg into mental health care may lead to better outcomes and overall well-being for patients. Oral Mg supplements may offer advantages in preventing depressive symptoms and supporting adjunctive therapy [112]. Additionally, Mg has been suggested as an adjuvant treatment for insomnia due to its role as a natural NMDA antagonist, GABA agonist, and relaxant [114].

Magnesium and osteoporosis

Magnesium Deficiency and Its Impact on Osteoporosis

Mg plays a crucial role in bone health, and its deficiency has been linked to the development of osteoporosis, a condition characterized by reduced bone mineral density (BMD) and increased risk of fractures [115]. Mg is involved in bone metabolism by regulating osteoblast and osteoclast activity, influencing bone formation and reabsorption [116]. Studies have shown that low Mg intake is associated with decreased BMD and increased risk of osteoporotic fractures [117]. Furthermore, Mg deficiency has been linked to impaired bone matrix formation and mineralization [118]. On the other hand, adequate Mg intake has been associated with improved bone health and reduced risk of osteoporosis. Mg supplementation has shown potential benefits in increasing BMD and reducing fracture risk, particularly in postmenopausal women [119].

Additionally, Mg interacts with other minerals, such as Ca and vitamin D, which are also essential for bone health. It is involved in the regulation of Ca homeostasis and the activation of vitamin D, further highlighting its importance in maintaining bone integrity [120]. Epidemiological studies have consistently demonstrated a positive correlation between elevated dietary Mg intake and BMD, while inadequate Mg intake has been associated with increased bone loss, particularly in postmenopausal osteoporotic women [121,122]. For instance, the findings from a study in 2024 revealed an increase in compressive stress and fracture toughness as the Mg concentration in the composition increased [123]. Moreover, selective dietary Mg deprivation led to the development of osteoporosis, characterized by increased skeletal fragility, enhanced bone reabsorption, diminished bone formation, and impaired bone growth [124,125].

Magnesium Deficiency and Its Impact on Mental Health

The mechanism behind these bone alterations may involve elevated concentrations of inflammatory cytokines, although the exact pathophysiological link remains unclear. Rude and Gruber demonstrated that Mg-deficient rats exhibited increased osteoclastic bone reabsorption accompanied by elevated levels of inflammatory substances such as substance P and TNF-α in bone tissue [126]. Additionally, Mg plays a crucial role in vitamin D synthesis, transport, and activation. Therefore, Mg deficits may impair the production of the active form of vitamin D, 1,25-dihydroxyvitamin D3 (1,25-(OH)2D3), leading to resistance to PTH and vitamin D actions [127]. This combined effect of Mg deficiency, altered PTH responsiveness, and reduced 1,25-(OH)2D3 synthesis can impair the bone formation and mineralization processes, ultimately diminishing bone quality, strength, and BMD.

Therapeutic Potential of Magnesium Supplementation

**Figure 4:**
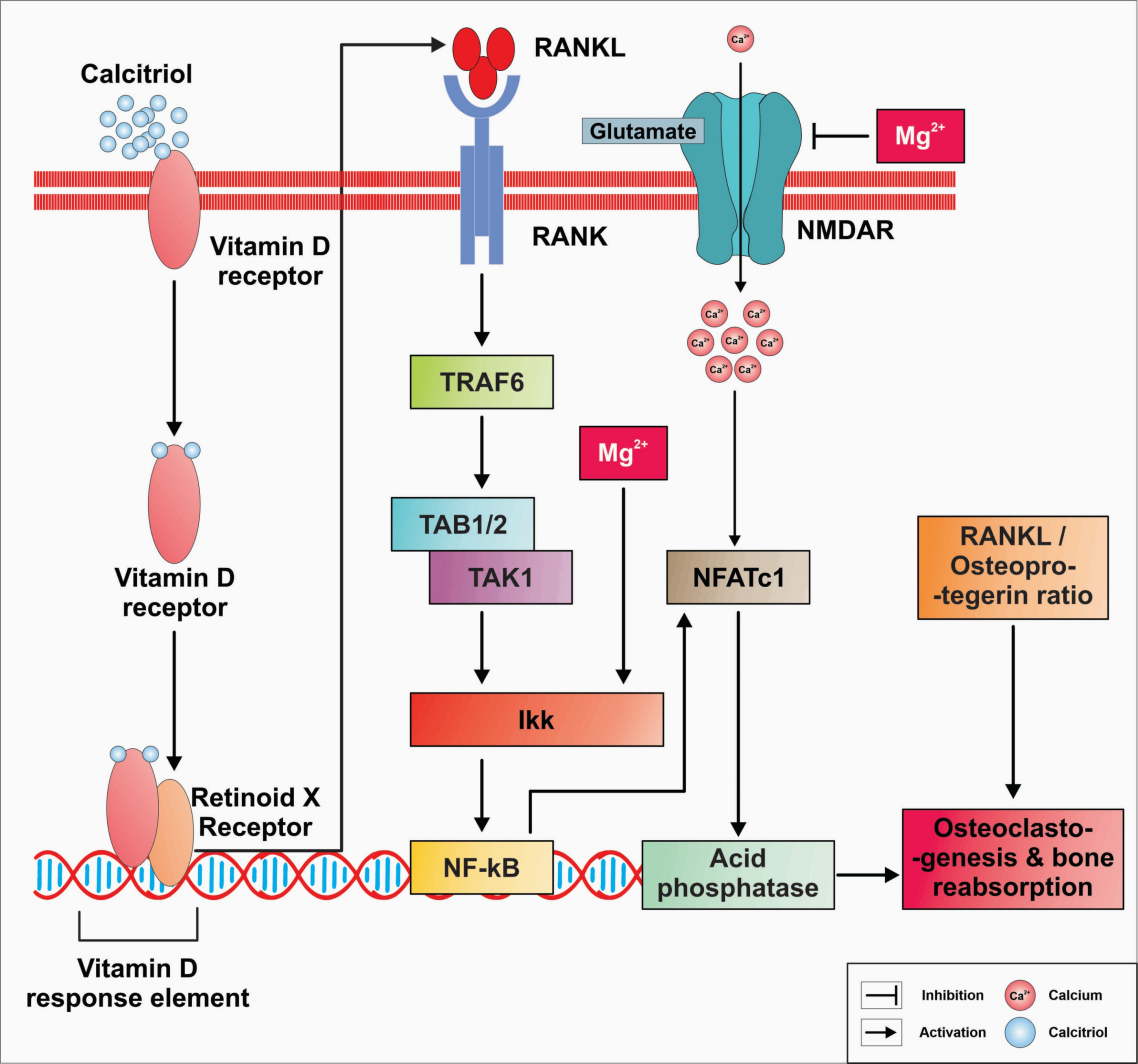
Figure describes various aspects of bone metabolism, including the regulation of bone formation and reabsorption, as well as the signaling pathways involved in these processes In the context of osteoporosis, inadequate intake of Mg or low Mg levels in the body can contribute to the upregulation of RANKL. RANKL regulates the survival, proliferation, and differentiation of osteoclasts by binding to its receptor, which is present in osteoclasts. The inducer of osteoclastogenesis, namely IKK expression, is regulated by Mg. Therefore, the dual role of Mg highlights its importance in maintaining balance in the human body. Mg, magnesium

Mg supplementation may improve bone health and reduce the risk of osteoporosis by enhancing Ca absorption, promoting bone mineralization, and supporting the activity of osteoblasts. Adequate Mg levels are essential for maintaining bone density and overall skeletal integrity in older adults. It has been hypothesized that Mg supplementation at doses sufficient to restore normal bone turnover may attenuate bone loss and reduce the risk of osteoporosis [128,129]. Overall, Mg plays a critical role in bone metabolism, and its deficiency may contribute to the development of osteoporosis. Ensuring adequate Mg intake through diet or supplementation may be beneficial for maintaining bone health and reducing the risk of osteoporotic fractures (Figure 4).

Magnesium and muscle health

Magnesium Deficiency and Its Impact on Muscle Health

Mg is crucial for muscle health, playing essential roles in muscle function, contraction, and relaxation [130]. Mg regulates Ca homeostasis, a fundamental process in muscle physiology. During muscle contraction, Mg blocks Ca channels, preventing excessive influx of Ca ions into muscle cells and thus promoting relaxation [131]. Studies have linked Mg deficiency to muscle weakness, cramps, and impaired muscle performance [132]. Low Mg levels can lead to increased neuromuscular excitability, resulting in hypercontractility and muscle spasms [133]. Mg deficiency has also been associated with an increased risk of muscle injuries and impaired muscle recovery following exercise [134]. Moreover, Mg plays a role in energy metabolism, as it is a cofactor for ATP production in muscle cells [135]. Adequate Mg levels are necessary for optimal energy production during muscle contraction and exercise performance [136]. Several studies have highlighted the importance of Mg supplementation in improving muscle function and performance.

Mechanisms Linking Magnesium on Muscle Health

Mg is essential for optimal muscle health, influencing various physiological mechanisms that promote muscle function, performance, and recovery. One of the primary roles of Mg is its involvement in muscle contraction and relaxation. It helps regulate Ca levels within muscle cells, which is crucial for initiating muscle contractions. When Mg levels are sufficient, Ca can enter muscle cells to trigger contraction, while Mg facilitates relaxation by counteracting Ca's effects.

Additionally, Mg is vital for the synthesis of ATP, the energy currency required for muscle activity. Adequate Mg levels ensure that muscles have the necessary energy to perform efficiently during physical activity and exercise. Moreover, Mg plays a role in protein synthesis, supporting muscle repair and growth. The mineral also has anti-inflammatory properties that can help reduce muscle soreness and damage following intense exercise. By minimizing inflammation, Mg aids in quicker recovery times, allowing athletes and active individuals to maintain performance levels and reduce the risk of injury. Furthermore, Mg supports neuromuscular function, influencing the transmission of signals between nerves and muscles, which is essential for coordination and strength. Collectively, these mechanisms highlight Mg's critical role in maintaining muscle health and enhancing physical performance.

Therapeutic Potential of Magnesium Supplementation

Mg plays a vital role in maintaining muscle health and function. As an essential mineral, it contributes to muscle contraction and relaxation by regulating Ca levels within muscle cells. Adequate Mg is necessary for the synthesis of ATP, the energy currency of the cell, which is crucial for muscle performance and endurance. Supplementation with Mg has been shown to alleviate muscle cramps, spasms, and fatigue, particularly in athletes and individuals engaged in strenuous physical activities. By improving muscle function, Mg can enhance exercise performance and recovery, reducing the risk of injury. Additionally, Mg's anti-inflammatory properties can help mitigate exercise-induced muscle damage and soreness, further supporting muscle recovery.

Research suggests that Mg may also play a role in preventing muscle loss associated with aging (sarcopenia) by promoting muscle protein synthesis. Older adults often experience lower Mg levels, contributing to decreased muscle mass and strength. Mg supplementation has been shown to reduce muscle cramps, enhance muscle strength, and improve exercise tolerance [137,138]. Furthermore, Mg supplementation may aid in the prevention and treatment of conditions such as delayed-onset muscle soreness (DOMS) [139]. Severe Mg deficits have been implicated in symptoms such as weakness, muscle pain, and night cramps. Study suggests that Mg deficiency may contribute to the development of fibromyalgia [140]. However, data on the effects of Mg supplements in alleviating fibromyalgia symptoms are limited, although there is some indication that Mg supplementation may help reduce tenderness, pain, and overall symptom severity in individuals with fibromyalgia [141]. In animal studies, dietary Mg deficiency in rats has been shown to increase the production of free radicals in skeletal muscle and may lead to various alterations in muscle cell metabolism, as well as structural impairments affecting the production of muscle energy necessary for contraction and relaxation [142]. A study by van Dronkelaar, 2023, demonstrated a significant and independent relationship between serum Mg levels and muscle performance, as well as several muscle parameters [143].

Additionally, another study found that Mg supplements (up to 8 mg/kg daily) improved muscle strength, endurance, and performance, and reduced oxygen consumption in young volunteers [144]. Furthermore, Veronese et al. showed that oral Mg supplementation (300 mg/day) improved physical performance, particularly in older individuals with a baseline low Mg dietary intake, suggesting that Mg supplementation may help prevent or delay age-related declines in physical performance [145]. Maintaining adequate Mg levels is essential for optimal muscle health and function. Mg deficiency can impair muscle contraction, increase the risk of muscle injuries, and hinder muscle recovery. Therefore, ensuring sufficient Mg intake through diet or supplementation is important for overall muscle health and performance. Therefore, Mg supplementation could be beneficial for preserving muscle health and function throughout the aging process. Mg supplementation presents a therapeutic opportunity for improving muscle health, enhancing athletic performance, and mitigating age-related muscle decline. Ensuring adequate Mg intake can significantly contribute to overall muscle well-being and functional capacity.

Specific dosage information of magnesium

Mg is an essential mineral that plays a crucial role in various bodily functions and is linked to the prevention and management of several health conditions. The RDA for Mg varies by age and gender but generally ranges from 310 to 420 mg per day for adults. Specific dosages can vary based on individual needs, conditions, and dietary intake.

Cardiovascular Diseases

In the context of cardiovascular health, research suggests that a daily intake of 300-600 mg of Mg may help reduce the risk of hypertension and CVD. Studies have shown that supplementation with Mg can lower blood pressure and improve overall heart health. It is important to consult with a healthcare provider for personalized dosage, especially for those on medications for heart conditions.

Migraines

For migraine prevention, a dosage of 400-600 mg of Mg per day has been found to be effective in reducing the frequency and severity of migraine attacks. Some studies recommend starting with a lower dose, around 200 mg per day, and gradually increasing it to assess tolerance and effectiveness. Mg citrate or Mg glycinate are often preferred forms due to their higher bioavailability.

Type 2 Diabetes

Individuals with type 2 diabetes may benefit from Mg supplementation in the range of 300-600 mg per day. Mg plays a critical role in glucose metabolism and insulin sensitivity. Some studies have shown that supplementation can improve fasting blood glucose levels and HbA1c, making it a valuable adjunct in diabetes management.

Mental Health

For mental health benefits, particularly in reducing symptoms of anxiety and depression, Mg dosages ranging from 200 to 400 mg per day have been studied. Mg supplementation may enhance mood by modulating neurotransmitter activity and improving sleep quality, both crucial for mental well-being.

Osteoporosis

In terms of osteoporosis prevention and management, an intake of 400-800 mg of Mg daily is recommended to support bone health. Mg is vital for Ca metabolism and bone density maintenance, and sufficient Mg levels are linked to lower rates of osteoporosis, particularly in postmenopausal women. While Mg is beneficial for various health conditions, individual needs can vary significantly. It is essential to consult with a healthcare professional before starting any supplementation regimen to determine the appropriate dosage based on personal health status and dietary intake. Additionally, obtaining Mg through a balanced diet rich in leafy greens, nuts, seeds, whole grains, and legumes is recommended alongside any supplementation.

Duration of magnesium supplementation and influence on health outcomes

The duration of Mg supplementation can significantly influence health outcomes in various conditions. Here is an overview of how long participants are typically supplemented in studies focused on CVDs, migraines, type 2 diabetes, mental health, and osteoporosis, along with how this duration may impact the results.

Cardiovascular Diseases

In studies examining Mg's effects on cardiovascular health, supplementation often lasts between three to 12 months. Longer durations can lead to more significant improvements in blood pressure and heart function, as Mg's role in vascular health may take time to manifest. Continuous supplementation is essential for maintaining optimal Mg levels, which can help sustain benefits over time.

Migraines

Research on Mg for migraine prevention usually involves supplementation for 12 weeks to six months. Short-term studies (around 12 weeks) have shown that participants can experience a reduction in the frequency and severity of migraine attacks. However, longer durations may provide more sustained relief and help establish long-term benefits. Continued supplementation can help prevent migraine recurrences by addressing underlying Mg deficiencies.

Type 2 Diabetes

Participants in studies assessing Mg’s role in managing type 2 diabetes are typically supplemented for three to six months. Research indicates that improvements in insulin sensitivity and blood glucose levels may occur within this timeframe. Extended supplementation may lead to more pronounced effects on metabolic markers, enhancing the overall management of diabetes and potentially preventing complications.

Mental Health

In mental health studies, Mg supplementation generally lasts four to 12 weeks, with some trials extending up to six months. Short-term studies have indicated improvements in anxiety and depressive symptoms. Longer durations may be beneficial for individuals with chronic mental health issues, as the cumulative effects of Mg on neurotransmitter modulation can enhance mood stabilization and overall mental well-being.

Osteoporosis

Mg supplementation for osteoporosis prevention typically spans six months to two years. This extended duration is crucial for observing significant changes in BMD and overall skeletal health. Mg's role in Ca metabolism and bone formation means that sustained supplementation can help strengthen bones and reduce the risk of fractures. The duration of Mg supplementation can greatly influence health outcomes by allowing sufficient time for the mineral to exert its physiological effects. Longer supplementation periods tend to yield more significant and sustained health benefits, particularly in chronic conditions. However, individual responses can vary, and factors such as baseline Mg levels, dietary intake, and the presence of comorbidities should also be considered. Regular monitoring and consultation with healthcare professionals are essential to optimize Mg supplementation and its associated health benefits.

Magnesium’s potential benefits in preventing cardiovascular diseases, migraine, type 2 diabetes, and osteoporosis

Mg is a vital mineral that plays a significant role in preventing and managing various health conditions, including CVDs, migraine, type 2 diabetes, and osteoporosis.

In the realm of cardiovascular health, Mg is crucial for maintaining normal heart rhythm, regulating blood pressure, and supporting overall heart function. Studies have shown that higher Mg intake is associated with a reduced risk of hypertension and coronary artery disease. This mineral helps relax blood vessels, which can lead to improved circulation and decreased risk of heart-related issues.

For individuals suffering from migraine, Mg has been recognized as an effective preventive treatment. Research indicates that Mg deficiency may trigger migraine attacks, and supplementation can reduce the frequency and severity of these episodes. By modulating neurotransmitter release and stabilizing neuronal membranes, Mg may help alleviate migraine symptoms.

Type 2 diabetes is another condition where Mg's role is increasingly being acknowledged. Adequate Mg levels are associated with improved insulin sensitivity, which is crucial for glucose metabolism. Low Mg intake has been linked to an increased risk of developing type 2 diabetes, and studies suggest that Mg supplementation may help lower blood sugar levels and improve metabolic health, thereby aiding in diabetes management.

Osteoporosis, a condition characterized by weakened bones and increased fracture risk, also benefits from Mg's influence. Mg is essential for Ca metabolism and bone density maintenance. Research indicates that adequate Mg intake can enhance bone mineralization and reduce the risk of osteoporosis, particularly in postmenopausal women who are at a higher risk for bone density loss. Mg is a multifunctional mineral with considerable benefits in preventing and managing conditions such as CVDs, migraines, type 2 diabetes, and osteoporosis. Ensuring sufficient Mg intake through diet or supplementation can be a key strategy in promoting long-term health and well-being.

Summary

This article highlights the critical importance of Mg in maintaining overall health and its role in preventing and managing various diseases. As an essential mineral, Mg is involved in over 300 enzymatic processes, supporting cardiovascular health by regulating blood pressure and heart rhythm. It plays a significant role in preventing migraines, with supplementation shown to reduce the frequency and severity of attacks. Additionally, Mg enhances insulin sensitivity, making it vital in managing type 2 diabetes and improving metabolic health. The mineral also contributes to mental well-being, as adequate Mg levels are associated with reduced symptoms of anxiety and depression. Furthermore, Mg is essential for bone health, aiding in Ca metabolism and reducing the risk of osteoporosis, particularly in postmenopausal women. The effectiveness of Mg supplementation varies by duration; studies often recommend periods ranging from a few weeks to several months to achieve optimal health outcomes. Overall, ensuring sufficient Mg intake through diet or supplementation is crucial for fostering long-term health, enhancing physical and mental well-being, and preventing chronic diseases. Regular monitoring and consultation with healthcare professionals can help optimize Mg levels for individual health needs.

## Conclusions

Mg plays a critical role in maintaining various physiological functions, such as energy production, enzyme activation, and cellular signaling. Deficiency in Mg is increasingly linked to conditions like cardiovascular diseases, diabetes, psychiatric issues, and musculoskeletal disorders, making it essential to maintain adequate levels. While dietary intake is important, the benefits of Mg supplementation particularly in older adults, where deficiency is common, need further exploration. Chronic Mg insufficiency has been suggested as a contributor to inflammation in aging, specifically the low-grade inflammation tied to aging and related diseases. Future research should focus on identifying effective Mg supplementation strategies to promote healthy aging and prevent disease.
